# Book Review: *My Bodyguard Brain – How Your Brain Uses Pain to Protect You*

**DOI:** 10.5334/cie.31

**Published:** 2021-06-15

**Authors:** Huub Vossen

**Affiliations:** 1 Heliomare, NL

**Keywords:** pain management, pain education, educator resource, paediatric medical conditions, paediatric pain, chronic pain, episodic pain, school issues, central sensitisation, adolescence pain, nociceptive plasticity, education, fear of pain, anxiety, depression, worry associated with pain, chronic stress, post-traumatic stress, child disability, epigenetic mechanisms, cognitive behavioural therapy

## Abstract

Review of a practical resource providing children with ongoing pain (and their parents) information of the neurobiology of pain. The book ‘My Bodyguard Brain – How your brain uses pain to protect you’ explains why we feel pain. The brain makes pain when it notices any sort of danger. Pain is associated with acute injury but it can also be evoked by social interaction and unpleasant emotion that children don’t know how to deal with. The text and drawings explain in a child-friendly way how your ‘Bodyguard’ brain wants to look after you, and how it sometimes gets a bit ‘too good’ at that job. It is a book I would recommend for teachers and clinicians dealing with children experiencing chronic pain. Ongoing pain in children is a huge problem in society. Paediatric pain should matter to everyone. It affects approximately one quarter to one third of all children and adolescents. Children with chronic pain have often cut back all their normal activities like school, sports, social life and sleep. Almost 60% of these children become adults experiencing pain and 50% of the children with pain has a parent suffering from chronic pain. Understanding what you feel will turn the oversensitive alarm down and is the first step to improve the lives of children and adolescents with pain.

“Review of a practical resource on the neurobiology of pain to children with ongoing pain and their parents.”

The book *My Bodyguard Brain – How Your Brain Uses Pain to Protect You* explains why we feel pain. Pain is never fun, but it is nevertheless very useful. Without our brain to guard our bodies, we wouldn’t feel any pain. If pain wasn’t unpleasant, you’d probably not even notice it and you might even carry on playing. But when something hurts, you’ll be careful for a while so that it has time to get better. Any pain you feel anywhere in your body is produced in your brain.

The ideas for the renewed evidence-based view on pain came from the researchers and pain scientists David Butler and Lorimer G. Moseley (Butler & Moseley, 2013, 2017).

The brain is like a bodyguard; it makes pain when it notices any sort of danger. That danger can be in your body such as in the case of a physical illness like appendicitis, for example, but it can also be caused by an emotional factor. No matter what, if your brain thinks it needs to protect you, it makes pain (Butler & Moseley, 2013, 2017). The brain can also create a tired feeling in your body or make you feel cold.

If you feel worried, sad, or angry and you can’t do much about it, you may start to feel pain. Your pain is real. You’re not making it up, and you’re not imagining it. Your brain is responsible for warning you, so it makes you feel pain in your body. Science has taught us that thoughts, emotions, and behaviour are important for the brain in terms of making pain or not making pain. These things are just as important as the signals that come from within our bodies. The brain can make you feel pain even when there’s nothing wrong with your body (Van de Kolk, 2015).

Once children and their families understand this concept, it’s easier to work towards getting the children (and their reassured parents) to be more active. This is important as movement is essential. Children (and parents) need to learn strategies to turn the oversensitive alarm down. That is why *My Bodyguard Brain* also includes a chapter with information for caregivers, parents, family members, and other people involved. Understanding what you feel is the first step to recovery.

Ongoing pain in children is a huge problem in society. It affects approximately one quarter to one third of all children and adolescents (Perquin et al., 2001). Pain that exists for any length of time, whether it is abdominal pain, a headache, or musculoskeletal pain, may become incapacitating because the pain has become the disease. The pain is absolutely real and has become a neurologic problem.

Parent protective responses are associated with maladaptive child outcomes. Parents who catastrophize their child’s pain (such as “My child’s pain is terrible, and it will never get better”) are more likely to experience a heightened level of distress, which in turn contributes to the child’s greater protective responses, increased pain intensity and disability (Neville et al., 2020).

Children with chronic pain often cut back all their normal activities like school, sports, social life, and sleep. Almost 60% of these children become adults experiencing pain. and 50% of the children with pain have a parent suffering from chronic pain (Logan et al., 2012; Walker et al., 2012).

The text and drawings in this book explain in a child-friendly way how your brain wants to look after you, how it works like a sort of bodyguard, and how it sometimes gets a bit “too good” at that job. This book is very easy to read and full of information about pain, presented in an understandable and illustrative style for both children and their parents.

In addition to its primary readers – children and their parents – I would recommend this book for therapists dealing with chronic pain for children and also for adults.

Rumping, K., & Bakkum van, R. (2020). *My bodyguard brain – How your brain uses pain to protect you*. Drukwerkconsultancy.

Website: ***www.mybodyguardbrain.com*** Email: ***mybodyguardbrain@gmail.com***
